# Clinical Manifestations and Outcomes of Renal Transplantation Patients With *Pneumocystis jirovecii* Pneumonia and Cytomegalovirus Co-infection

**DOI:** 10.3389/fmed.2022.860644

**Published:** 2022-04-11

**Authors:** Jilin Zou, Tao Qiu, Jiangqiao Zhou, Tianyu Wang, Xiaoxiong Ma, Zeya Jin, Yu Xu, Long Zhang, Zhongbao Chen

**Affiliations:** Department of Organ Transplantation, Renmin Hospital of Wuhan University, Wuhan, China

**Keywords:** *Pneumocystis jirovecii* pneumonia, renal transplantation, clinical outcome, cytomegalovirus infection, impact

## Abstract

**Background:**

*Pneumocystis jirovecii* pneumonia (PJP) and cytomegalovirus (CMV) infection are common opportunistic infections among renal transplantation (RT) recipients, and both can increase the risk of graft loss and patient mortality after RT. However, few studies had evaluated PJP and CMV co-infection, especially among RT patients. Therefore, this study was performed to evaluate the impact of CMV co-infection with PJP among RT recipients.

**Methods:**

We retrospectively analyzed the clinical data of patients with confirmed diagnosis of PJP between 2015 and 2021 in our hospital. We divided patients into PJP and PJP+CMV groups according to their CMV infection status, and the clinical severity and outcomes of the two groups were evaluated.

**Results:**

A total of 80 patients after RT were diagnosed with PJP. Of these, 37 (46.2%) patients had co-existing CMV viremia. There were no statistically significant intergroup differences in age, sex, diabetes, onset time of PJP after RT and postoperative immunosuppressant. Compared to serum creatinine (Cr) at admission, the serum Cr at discharge in both the PJP and PJP+CMV groups were decreased. The PJP+CMV group had a higher C-reactive protein level, higher procalcitonin level, and lower albumin level than the PJP group. The PJP+CMV group showed a higher PSI score than the PJP group. Moreover, the initial absorption time of the lesion was longer in the PJP+CMV group. However, the duration of hospitalization showed no significant differences between the two groups. The mortality rate was 9.4-times higher in the PJP+CMV group than in the PJP group. The rate of admittance to the intensive care unit was 3.2-times higher in the PJP+CMV group than in the PJP group.

**Conclusion:**

CMV co-infection may result in more serious inflammatory response. RT patients with PJP+CMV infection had more severe clinical symptoms, slower recovery from pneumonia, and higher mortality than those with PJP alone. Therefore, when RT patients present with severe PJP, the possibility of CMV co-infection should be considered. Short-term withdrawal of immunosuppressants in case of severe infection is safe for the renal function of RT patients.

## Introduction

Owing to the use of immunosuppressants, post-renal transplantation (RT) patients are recognized as having high risk of opportunistic infection. Among them, *Pneumocystis jirovecii* pneumonia (PJP), caused by *P. jirovecii*, is one of the most common infections after RT and is life-threatening (1). The peak incidence period is within 6 months after RT. Besides PJP, cytomegalovirus (CMV) is also a common infection that can increase the risk of graft loss and patient mortality after solid organ transplantation (2, 3). In the general population, CMV is mostly latent infection after primary infection in childhood. The CMV seroprevalence rate reportedly between 30 and 97% depending on geography and socioeconomic status (4, 5). In RT recipients, CMV infection often occurs during the first 3 months after RT by reactivation of latent infection. Several previous studies suggested that PJP is often co-infection with CMV followed by devastating clinical consequences in immunocompromised non-HIV patients or solid organ transplantation recipients (6–9). But the impact of co-infection of CMV on clinical manifestations and outcomes of PJP patients after deceased donor RT has not been well determined. Therefore, we retrospectively analyzed the clinical characteristics and outcomes of 80 patients confirmed with PJP after deceased donor RT and assessed the impact of CMV co-infection on the clinical severity and outcomes and laboratory tests for PJP patients after RT.

## Materials and Methods

### Patients and Methods

A total of 1,300 RT recipients were followed-up in our hospital between 2015 and 2021. Eighty patients confirmed with PJP were included in this retrospective study. The Medical Research Ethics Committee of the Renmin Hospital of Wuhan University approved the study. The hospital is committed to protecting patient privacy and adhered with the tenets of the Helsinki Declaration. The clinical data of the 80 patients, such as age, sex, RT operation time, induction of immunosuppressive agents, clinical symptoms, lung CT, duration between PJP and transplantation surgery, laboratory test results at admission and discharge, mean hospitalization days, initial absorption time of the lesion, pneumonia severity index (PSI) score, and treatment outcome were all collected. The patients were divided into two groups as follows: 43 patients with PJP as the PJP group and 37 patients with PJP and co-infection with CMV as the PJP+CMV group. Immunosuppression after RT was maintained by mycophenolate mofetil (MMF) combined with tacrolimus or cyclosporine A and glucocorticoids. The plasma concentration of tacrolimus was maintained at 6–8 μg/L, while that of cyclosporine A was maintained at 150–200 μg/L. The diagnosis of PJP was confirmed based on the following criteria. (1) Fever and respiratory symptoms such as cough, dyspnea; (2) Microbial pathogen diagnosis: detection of *P. jirovecii* in sputum samples or peripheral blood using metagenomic next-generation sequencing (mNGS). (3) Clinical diagnosis: This was based on clinical features, typical CT imaging presentment, elevated 1,3-β-D-glucan levels, and condition improved after anti-PJP therapy. All patients received CMV serological detection before renal transplantation. And CMV DNA in whole blood was detected once a month after renal transplantation. CMV viremia was defined as CMV DNA PCR titer ≥500 copies/mL in whole blood. Lung CT was performed every 7 days after admission, and the initial absorption time of the lesion was defined as the duration from hospital admission to the time that the lung lesions started to show some improvement.

### Statistical Analysis

IBM SPSS software package (version 20.0; IBM Corporation, Armonk, NY, USA) was used for statistical analysis. Data were described using mean, standard deviation, median, and numbers (percentages). The chi-square and two-tailed *t*-test were performed to compare group differences. In addition, paired-samples *t*-test of serum creatinine (Cr) between patients at admission and discharge was performed in the PJP and PJP+CMV groups. *P* < 0.05 was considered as being statistically significant.

## Results

### Comparison of Demographic Characteristics Between PJP and PJP+CMV Groups

As of December 2021, a total of 80 patients after RT were diagnosed with PJP: 37 (46.2%) patients had co-existing CMV viremia. All the patients received RT from deceased donor, and did not have other pathogenic infections. The PJP-prophylaxis protocol consisted of trimethoprim–sulfamethoxazole (trimethoprim 40 mg, sulfamethoxazole 200 mg) daily for all recipients after RT. According to the individual condition, the duration of PJP prophylaxis varied from 3 to 6 months after RT. Remarkably, one month prior to PJP infection, all recipients already did not receive trimethoprim–sulfamethoxazole prophylaxis. In addition, the CMV-prophylaxis protocol consisted of ganciclovir (1.5 g/d, divided into three doses) daily for all recipients during the first 3 months after RT. As shown in Table 1, there were no statistically significant intergroup differences in age, sex, diabetes, and postoperative immunosuppressant. The average age was 42.4 ± 9.7 years in the PJP group and 45.5 ± 10.6 years in the PJP+CMV group. Most of the maintenance immunosuppressant regimen was MMF combined with calcineurin inhibitor (CNI) and steroids. The onset time of PJP after RT between the two groups was similar (8.1 ± 5.0 months vs. 12.8 ± 16.6 months; *P* = 0.106; Table 1). And the history of acute rejection, between the two groups was also similar (41.9 vs. 43.2%; *P* = 0.901; Table 1). The proportion of onset time ≤ 6 months, 6–12 months, and ≥12 months was not different between the two groups. The use of ATG prior to PJP was also similar between the PJP and PJP+CMV groups.

**Table 1:** Comparison of demographic characteristics between the PJP and PJP+CMV groups.

**Characteristic**	**PJP group** **(*n* = 43)**	**PJP+CMV group (*n* = 37)**	***P* value**
Age (years), mean ± SD	42.4 ± 9.7	45.5 ± 10.6	0.188
Sex			
Male, *N* (%)	27 (62.8%)	27 (73%)	0.332
Diabetes, *N* (%)	1 (2.3%)	1 (2.7%)	0.914
Postoperative immunosuppressant			
MMF+tacrolimus+prednisolone, *N* (%)	42 (97.7%)	35 (94.6%	0.47
MMF+cyclosporine+prednisolone, *N* (%)	1 (2.3%)	2 (5.4%)	
Onset time (month, mean ± SD)	8.1 ± 5.0	12.8 ± 16.6	0.106
≤ 6 months, *N* (%)	20 (46.5%)	20 (54.1%)	0.453
6–12 months, *N* (%)	16 (37.2%)	9 (24.3%)	
≥12 months, *N* (%)	7 (16.3%)	8 (21.6%)	
Use of ATG prior to PJP, *N* (%)	16 (37.2%)	13 (35.1%)	0.847
History of acute rejection, *N* (%)	18 (41.9%)	16 (43.2%)	0.901
Under prophylaxis following RT, *N* (%)	43 (100%)	37 (100%)	1
Under prophylaxis at diagnosis, *N* (%)	0	0	1

### Comparison of Laboratory Tests Between PJP and PJP+CMV Groups

As shown in Table 2, there were no statistically significant differences in 1,3-β-D-glucan levels, serum Cr at admission, serum Cr at discharge, white blood cell count, and lymphocyte count between the two groups. The PJP+CMV group had a higher C-reactive protein (CRP) level, lower albumin (ALB) level, and higher procalcitonin (PCT) level than the PJP group (*P* = 0.024, *P* = 0.006, *P* = 0.009, respectively). Moreover, paired-samples *t*-test of serum Cr between patients at admission and discharge was also performed in the PJP group and PJP+CMV group. Compared to serum Cr at admission, the serum Cr at discharge in both the PJP and PJP+CMV groups were decreased.

**Table 2:** Comparison of laboratory findings and outcome between the PJP and PJP+CMV groups.

**Variable**	**PJP group** **(*n* = 43)**	**PJP+CMV group** **(*n* = 37)**	***P* Value**
Serum Cr at admission (mg/dL), mean ± SD	159.6 ± 74.1	186.2 ± 99.8	0.176
Serum Cr at discharge (mg/dL), mean ± SD	126.2 ± 40.0^†^^#^	140.7 ± 58.2^†^^#^	0.217
1,3-β-D-glucan (pg/mL), mean ± SD	664 ± 621	659 ± 561	0.974
C-reactive protein (mg/L), mean ± SD	61.2 ± 34.4	87.2 ± 53.7	0.024^*^
Procalcitonin (ng/mL), mean ± SD	0.22 ± 0.33	0.56 ± 0.67	0.009^*^
White Blood Cell (10^9^/L), mean ± SD	8.1 ± 3.9	8.2 ± 3.9	0.975
Lymphocyte (10^9^/L), mean ± SD	0.7 ± 0.4	0.6 ± 0.3	0.147
Albumin (g/L), mean ± SD	38.4 ± 4.0	35.9 ± 3.7	0.006^*^
Initial absorption time of lesion (days), mean ± SD	12.9 ± 5.8	20 ± 7.1	0.000^*^
Hospitalization days, mean ± SD	27.7 ± 12.7	32.6 ± 11.5	0.079
PSI score			
Class I -III, N (%)	12 (27.9%)	1 (2.7%)	0.002^*^
Class IV, N (%)	28 (65.1%)	19 (51.4%)	0.212
Class V, N (%)	3 (7.0%)	17 (45.9%)	0.000^*^
Death, N (%)	1/43 (2.3%)	8/37 (21.6%)	0.006^*^
Admitted to ICU, N (%)	4/43 (9.3%)	11/37 (29.7%)	0.02^*^

* *P < 0.05 was considered statistically significant.*

† *Data excluded the death cases.*

# *Paired-samples t-test of serum Cr between patients at admission and discharge was performed in the PJP group and PJP+CMV group respectively. P < 0.05 was considered statistically significant*.

### Comparison of Pneumonia Severity and Hospitalization Between the PJP and PJP+CMV Groups

As shown in Table 2, according to the PSI score system, the proportion of class V(severe) in the PJP+CMV group was significantly higher than the PJP group. Moreover, the initial absorption time of the lesion was longer in the PJP+CMV group. However, the duration of hospitalization had no significant differences between the two groups.

### Comparison of Treatment and Clinical Outcomes Between the PJP and PJP+CMV Groups

The treatment between the two groups was similar. All patients discontinued the use of immunosuppressive agent for 1–2 weeks; trimethoprim–sulfamethoxazole (TMP–SMZ) (TMP 15–20 mg/kg and SMZ 75–100 mg/kg, divided into three doses) combined with caspofungin (50 mg/d, loading dosage 70 mg on the first day) were used as the first-line drugs, and methylprednisolone (40–120 mg/d) was used to control the inflammation and temperature. For patients with CMV viremia, ganciclovir was used as antiviral therapy with modified renal dose. As shown in Table 2, during the treatment period, a total of nine patients died (one patient in the PJP group and eight in the PJP+CMV group). The mortality rate was 9.4-times higher in the PJP+CMV group than in the PJP group (2.3 vs. 21.6%; *P* = 0.006). In addition, a total of 15 patients were admitted to the intensive care unit (ICU) (four patients in the PJP group and 11 in the PJP+CMV group). The rate of ICU admittance was 3.2-times higher in the PJP+CMV group than in the PJP group (9.3 vs. 29.7%; *P* = 0.02).

## Discussion

We conducted a retrospective review of the clinical characteristics and outcome of 80 patients confirmed with PJP to identify the impact of CMV co-infection on the clinical severity and outcomes and laboratory tests for PJP patients after RT. In this study, we observed that the incidence of CMV co-infection with PJP was 46.2% in deceased donor RT recipients. Similar to a previous study (9), we could show that the combined PJP and CMV co-infection increases the clinical severity in a larger cohort of age-matched PJP patients in deceased donor RT recipients, and the PJP+CMV group had a higher CRP level, lower ALB level, and higher PCT level than the PJP group. Serum ALB or CRP levels were considered good predictive markers for morbidity and mortality of critically ill patients (10–12). And the serum levels of CRP and PCT increase apparently in response to infection or tissue injury, the degree of increase was significantly correlated with the severity of infection, and the ALB level may exist decrease during the response to acute infections. Therefore, the higher CRP level and PCT level and lower ALB level in the PJP+CMV group may also be representative of more severe infection and serious inflammatory response.

Remarkably, in the present study, we clearly demonstrated that the rate of patients admitted to the ICU in the PJP+CMV group was 3.2-times higher than that in the PJP group and the rate of death was 9.4-times higher in the PJP+CMV group than in the PJP group, which was different from that previously reported (9). This may be related to the fact that all donors in this study were deceased and had a longer ischemic time and stronger immunosuppressive regimen than living donors, and the patients had a higher incidence of CMV co-infection. In addition, most PJP cases in our study were early onset, which had a stronger immunosuppressive state. Interestingly, the serum Cr of patients at discharge in the PJP and PJP+CMV groups were all lower than the time at admission. This further proves that in case of severe infection, short-term withdrawal of immunosuppressants will not affect the patient's renal function or increase the incidence of acute rejection. The renal function of patients may have improved with the discontinuation of tacrolimus. Moreover, during the present study period, no one had graft failure except the death-censored graft loss.

Previous studies suggested that CMV infection can suppress the function of antigen-presenting cells and helper T cells, and cause further immune suppression, which may lead to subsequent PJP infection and delay the recovery of PJP (13–16). Our findings further support their conclusions, as the initial absorption time of the lesion in the present study was longer in the PJP+CMV group than the PJP group. Therefore, patients with PJP and CMV co-infection showed delayed improvement from pneumonia. However, the duration of hospitalization had no significant differences between the two groups. This may be because the duration of hospitalization is more susceptible to other confounding factors. CMV is one of the most frequent viruses associated with RT (17). According to a previous study (18), 60% RT recipients may develop active infection and 20% developed CMV disease. In the general population, CMV infection is usually asymptomatic and considered relatively self-limiting. However, given the use of immunosuppressive agents, the risk of active CMV infection (including primary infection or reactivation of latent infection) or CMV syndrome or CMV disease (i.e., CMV pneumonitis, CMV colitis, CMV esophagitis, CMV gastritis, CMV hepatitis, and CMV nephritis) is increased in RT recipients. Remarkably, most CMV infections in our study involved reactivation rather than primary infection. Currently, because of the widespread use of prophylactic and preemptive antivirals, the incidence rate of CMV infection has greatly reduced in RT patients. However, about 25% of RT recipients may still develop CMV infection (19). Owing to the adverse effects such as leukopenia of ganciclovir or other drugs, many patients may reduce the dose of medications including antiviral prophylaxis, which leads to an increased risk of CMV infection (20). Therefore, monitoring the CMV reactivation is critical (21).

In the present study, the 37 patients with CMV co-infection received intravenous ganciclovir combined with TMP-SMZ and caspofungin as anti-pneumocystis therapy, while the patients with only PJP received TMP-SMZ and caspofungin treatment alone. However, the mortality rate and patients admitted to the ICU in the PJP+CMV group was still higher than that in the PJP group. We speculate that the reasons for higher rate of death in our cohort are as follows. First, the dose of ganciclovir may not be enough owing to the drugs' side effects and impaired renal function, and the clinical severity increased in the PJP+CMV group. Second, CMV reactivation could be an indicator of stronger immunosuppression status and illness severity. The degree of CMV viral load is strongly associated with the mortality of RT patients (22). Moreover, CMV viremia cannot exclude the possibilities of CMV pneumonia rather than only CMV reactivation. Furthermore, viral infections are controlled mainly by viral-specific T cells, and the clearance and prediction of CMV viremia is associated with the detection of CMV-specific T cells (23–26). Patients in the PJP+CMV group may have a lower CMV-specific T-cell count because of prior CMV infection.

Our study has some limitations. First, on account of being a single-center, retrospective and observational study, inherent bias is inevitable. Second, the numbers of patients in each group were still small; thus, additional prospective, multicenter, large-scale clinical studies are necessary to accurately evaluate the impact of CMV co-infection with PJP. Moreover, long-term follow-up was not performed in our study.

In conclusion, RT patients with PJP+CMV infection had more severe clinical symptoms, more serious inflammatory response, slower recovery from pneumonia, and higher mortality than PJP patients. Therefore, monitoring the CMV reactivation after RT are critical and when RT patients present with severe PJP, the possibility of CMV co-infection should be considered. In addition, short-term withdrawal of immunosuppressants in case of severe infection is safe for the renal function of RT patients. Further studies are needed to confirm underlying mechanisms.

## Data Availability Statement

The original contributions presented in the study are included in the article/supplementary material, further inquiries can be directed to the corresponding author.

## Ethics Statement

The studies involving human participants were reviewed and approved by the Medical Research Ethics Committee of the Renmin Hospital of Wuhan University. The patients/participants provided their written informed consent to participate in this study.

## Author Contributions

JZo and ZC contributed to the design of the study, as well as managing and interpreting the data, and drafting and revising the manuscript. JZo, TQ, JZh, TW, XM, ZJ, YX, and LZ contributed to the collection and interpretation of the data, and were responsible for statistical work. All authors have approved the manuscript and agree to be accountable, and contributed substantially in the process of completing this study and had full access to the data.

## Funding

This study was supported by the National Natural Science Foundation of China (Nos. 81870067 and 81400753).

## Conflict of Interest

The authors declare that the research was conducted in the absence of any commercial or financial relationships that could be construed as a potential conflict of interest.

## Publisher's Note

All claims expressed in this article are solely those of the authors and do not necessarily represent those of their affiliated organizations, or those of the publisher, the editors and the reviewers. Any product that may be evaluated in this article, or claim that may be made by its manufacturer, is not guaranteed or endorsed by the publisher.
